# Limited effect of lymph node status on the metastatic pattern in colorectal cancer

**DOI:** 10.18632/oncotarget.9064

**Published:** 2016-04-27

**Authors:** Nikki Knijn, Felice N. van Erning, Lucy I.H. Overbeek, Cornelis J.A. Punt, Valery E.P.P. Lemmens, Niek Hugen, Iris D. Nagtegaal

**Affiliations:** ^1^ Department of Pathology, Radboud University Medical Center, Nijmegen, The Netherlands; ^2^ Netherlands Comprehensive Cancer Organisation, Eindhoven, The Netherlands; ^3^ Department of Public Health, Erasmus MC University Medical Centre, Rotterdam, The Netherlands; ^4^ PALGA Foundation, Houten, The Netherlands; ^5^ Department of Medical Oncology, Academic Medical Center, University of Amsterdam, Amsterdam, The Netherlands; ^6^ Department of Surgery, Radboud University Medical Centerl, Nijmegen, The Netherlands

**Keywords:** colorectal neoplasms, neoplasm metastasis, lymph nodes, blood vessels, autopsy, Pathology Section

## Abstract

Regional lymph node metastases in colorectal cancer (CRC) decrease outcome. Whether nodal metastases function as a biomarker, i.e. as a sign of advanced disease, or are in fact involved in the metastatic process is unclear. We evaluated metastatic patterns of CRC according to the lymph node status of the primary tumor.

A retrospective review of 1393 patients with metastatic CRC who underwent autopsy in the Netherlands was performed. Metastatic patterns of regional lymph node positive and negative CRC were compared and validated by population-based data from the Eindhoven Cancer Registry (ECR).

Patients with regional lymph node positive CRC more often developed peritoneal metastases (28% *vs*. 21%, *p*=0.003) and distant lymph node metastases (25% *vs*. 15%, *p* <0.001). Incidences of liver and lung metastases were comparable. Data from the ECR confirmed our findings regarding peritoneal (22.4% *vs*. 17.0%, *p*=0.003) and distant lymph node metastases (15.8% *vs*. 9.7%, *p* <0.001).

Regional lymph node positive CRC show a slightly different dissemination pattern, with higher rates of peritoneal and distant lymph nodes metastases. Comparable incidences of liver and lung metastases support the hypothesis that dissemination to distant organs occurs independently of lymphatic spread.

## INTRODUCTION

Despite intensive follow-up and increasing therapeutic options for colorectal cancer (CRC), metastatic disease remains the leading factor in CRC mortality. CRC most frequently metastasizes to the liver, lung and peritoneum, but other metastatic sites such as bone, spleen, brain and distant lymph nodes have been described.[1–3]

According to the mechanistical view of metastatic spread, tumor cells can disseminate to distant organs through two pathways: the vascular and the lymphatic pathway. The vascular hypothesis suggest that blood vessels transport tumor cells directly to distant organs. In the lymphatic pathway tumor cells may disseminate from regional lymph nodes to distant lymph nodes, reach the systemic circulation and subsequently form organ metastases.[4] The distinction between these pathways and their role in dissemination remains matter of debate.

Post-mortem studies offer a possibility to register both the extent and location of metastatic disease. Findings during autopsy may be considered the ultimate endpoint of disease. Autopsy studies are therefore usefull for getting insight in the relevance of lymphatic spread in the dissemination of cancer. Most autopsy studies, have focused on metastatic patterns in one or more types of cancer, but have failed to address differentiating aspects such as lymph node involvement. A large autopsy study by Budczies et al. across major cancer types, showed higher rates of metastases in distant lymph nodes, peritoneal cavity, pleura, pericardial and adrenal glands in lymph node positive tumors.[5] Although this study has given insight into metastatic patterns, this was done by grouping various cancers together.

To gain insight in the relevance of lymphatic spread in the dissemination of CRC, we evaluated patterns of metastases according to the lymph node status of the primary tumor in 1393 autopsies. To confirm the clinical relevance, we analyzed population-based data from the Eindhoven Cancer Registry.

## RESULTS

In the autopsy cohort, there were 1393 patients with metastatic disease; 879 patients (63%) with regional lymph node metastases (N+) and 514 patients (37%) without regional lymph node involvement (N-). The distribution of patient and tumor characteristics according to regional lymph node status is presented in Table 1. N+ patients more often had a higher T-stage (T3/T4: 90.8% vs. 85.4%, p<0.001), a tumor located in the proximal part of the colon (38.3% vs. 31.5%, p=0.01), mucinous or signet ring cell histology (20.9% vs. 14.2%, p=0.002), multiple metastases (56.5% vs. 43.2%, p<0.001) and synchronous onset of metastases (61.2% vs. 36.2%, p<0.001).

**Table 1:** Distribution of tumor and patient characteristics according to regional lymph node status of the primary tumor in the autopsy cohort

Features	N+		N-		*P*-value
	879	(%)	514	(%)	
**Gender**					0.375
Male	514	58.5	313	60.9	
Female	365	41.5	201	39.1	
**Age at diagnosis**					0.681
<60	188	21.4	96	18.7	
60-74	408	46.4	247	48.1	
≥75	283	32.2	171	33.3	
**Location of primary**					0.032
Proximal colon	337	38.3	162	31.5	
Distal colon	271	30.8	162	31.5	
Rectum	203	23.1	135	26.3	
Colon, not specified	68	7.7	55	10.7	
**T Stage**					<0.001
T1	0	0	7	1.4	
T2	37	4.2	53	10.3	
T3	605	68.8	338	65.8	
T4	193	22.0	101	19.6	
Not specified	43	4.9	15	2.9	
**Histology**					0.002
Non-mucinous adenocarcinoma	695	79.1	441	85.8	
Mucinous adenocarcinoma	156	17.7	68	13.2	
Signet ring cell	28	3.2	5	1.0	
**Onset of metastases**					<0.001
Synchronous	538	61.2	186	36.2	
Metachronous	341	38.8	328	63.8	
**Number of distant metastases**					<0.001
1	382	43.5	292	56.8	
>1	497	56.5	222	43.2	

N+: primary tumor with regional lymph node metastases;

N-: primary tumor without regional lymph node metastases.

### Distribution of metastases

**Figure 1:**
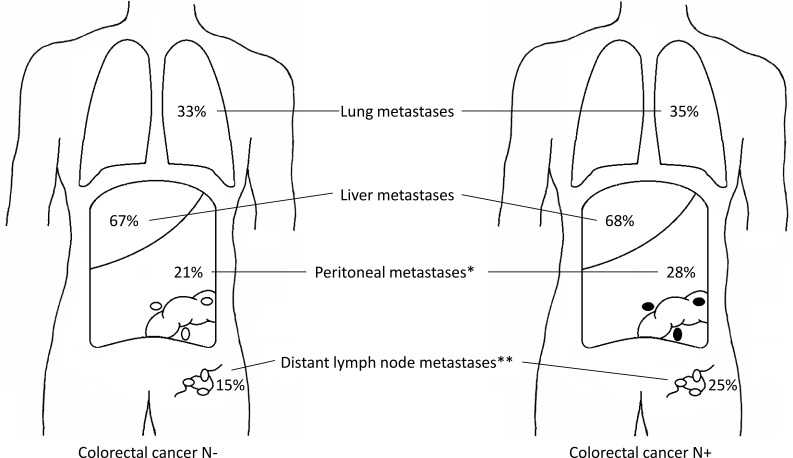

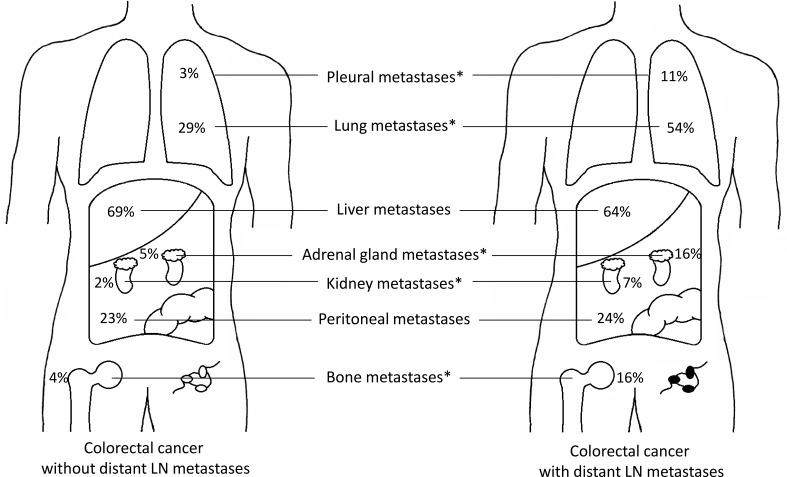
**a** Distribution of CRC metastases according to regional lymph node status in the autopsy cohort. Left figure shows the distribution of metastases for regional lymph node negative primary tumors, right figure shows the distribution of metastases for regional lymph node positive primary tumors. * *p* = 0.003, ** *p* < 0.001. **b**. Distribution of CRC metastases according to distant lymph node positivity in the autopsy cohort. Left figure shows the distribution of metastases for primary tumors without distant lymph node metastases, right figure shows the distribution of metastases for primary tumors with distant lymph node metastases. * *p* < 0.001.

The liver was the most frequent site of metastasis irrespective of regional lymph node status (68% in N+ and 67% in N-, p=0.53). Lung metastases occurred in 33% of N- patients and in 35% of N+ patients (p=0.42; Figure 1a, Supplementary Figure 1a). There was a higher rate of peritoneal metastases in N+ patients (28% vs. 21%, p=0.003) and distant lymph node metastases were more often found in N+ patients (25% vs. 15%, p<0.001). Other significant differences were found for metastases in omentum, spleen and pancreas (N+ vs. N-; 9.2 vs. 3.3%, p<0.001, 2.8 vs. 1.0%, p=0.02, and 2.6 vs. 1.0%, p=0.04, respectively).

When patients were subdivided according to the location of the primary tumor, a higher percentage of lung metastases in N+ rectal cancer as compared to N+ colon cancer (43.8% vs. 32.1%, p=0.001) and higher percentage of peritoneal metastases in N+ colon cancer as compared to N+ rectal cancer (30.8% vs. 19.2%, p=0.002) was found. This could be related to the higher overall percentage of lung metastases in rectal cancer compared with colon cancer (42.6% vs. 31.3%, p<0.001) and of peritoneal metastases in colon cancer compared with rectal cancer (28.1% vs. 17.2%, p<0.001). N+ colon cancers more often had peritoneal metastases and distant lymph node metastases compared with N- colon cancers (24.0% vs. 14.0%, p<0.001, and 30.8% vs. 23.2%, p=0.009, respectively). In rectal cancer only distant lymph node metastases were more often seen in N+ than in N- patients (28.1% vs. 18.5%, p=0.05).

### Validation of findings

A total of 2382 patients with metastatic colorectal cancer from the Eindhoven Cancer Registry (ECR) was included, of which 1711 patients (71.8%) had regional lymph node metastases and 671 patients (28.2%) did not. Median follow-up was 5.0 years (range 1.2–8.8 years). The distribution of patient and tumor characteristics according to lymph node status is presented in Table 2.

**Table 2:** Distribution of tumor and patient characteristics according to regional lymph node status of the primary tumor in the clinical cohort

Features	N+		N-		*P*-value
	1711	(%)	671	(%)	
**Gender**					0.583
Male	952	55.6	365	54.4	
Female	759	44.4	306	45.6	
**Age at diagnosis**					0.008
<60	448	26.2	135	20.1	
60-74	814	47.6	342	51.0	
≥75	449	26.2	194	28.9	
**Location of primary**					0.014
Proximal colon	609	35.6	192	28.6	
Distal colon	504	29.5	216	32.2	
Rectum	572	33.4	252	37.6	
Colon, unknown	26	1.5	11	1.6	
**T Stage**					<0.001
T1	7	0.4	12	1.8	
T2	83	4.8	89	13.3	
T3	1101	64.4	411	61.2	
T4	397	23.2	106	15.8	
Unknown	123	7.2	53	7.9	
**Histology**					0.230
Non-mucinous adenocarcinoma	1562	91.3	621	92.5	
Mucinous adenocarcinoma	121	7.1	45	6.7	
Signet ring cell	28	1.6	5	0.8	
**Onset of metastases**					<0.001
Synchronous	1155	67.5	318	47.4	
Metachronous	556	32.5	353	52.6	
**Number of distant metastases**					<0.001
1	1067	62.4	460	68.6	
>1	644	37.6	211	31.4	

N-: primary tumor without regional lymph node metastases;

N+: primary tumor with regional lymph node metastases

N+ patients more often developed peritoneal metastases and distant lymph node metastases (22.4% vs. 17.0%, p=0.003, and 15.8% vs. 9.7%, p<0.001). Moreover, there was a higher percentage of liver metastases and a lower percentage of lung metastases in N+ patients compared with N- patients (71.6% vs. 66.3%, p=0.01 and 23.7% vs. 27.7%, p=0.04, respectively).

Logistic regression analyses identified several clinicopathological factors that were associated with location specific metastases (Table 3). Rectal tumors were associated with a higher risk of developing liver and lung metastases (OR for liver: 1.33 (1.05-1.67), p<0.05, OR for lung: 2.25 (1.74-2.89), p<0.001), and with a lower risk of developing peritoneal carcinomatosis (OR: 0.24 (0.18-0.32), p<0.001). T4 tumors less often led to liver metastases (OR: 0.48 (0.38-0.61), p<0.001) and were associated with a higher risk of developing peritoneal carcinomatosis (OR: 2.18 (1.72-2.77), p<0.001). Lymph node positive tumors were associated with an increased risk of developing distant lymph node metastases, especially N2 tumors (OR: 3.03 (2.14-4.29), p<0.001). N2 tumors were also associated with peritoneal carcinomatosis (OR: 1.40 (1.05-1.87), p<0.05). Mucinous tumors less often led to liver metastases (OR: 0.46 (0.34-0.62), p<0.001) and were associated with a higher risk of developing peritoneal carcinomatosis (OR: 2.53 (1.84-3.47), p<0.001).

**Table 3:** Risk of developing distant metastases in the clinical cohort

Clinicopathological factors	Risk of developing liver metastases	Risk of developing lung metastases	Risk of developing peritoneal carcinomatosis	Risk of developing distant lymph node metastases
	MV analyses OR (95% CI)	MV analyses OR (95% CI)	MV analyses OR (95% CI)	MV analyses OR (95% CI)
**Age**				
<59	1.00	1.00	1.00	1.00
60-74	0.99 (0.78-1.25)	1.26 (0.98-1.61)	0.85 (0.65-1.11)	0.87 (0.65-1.16)
≥75	0.96 (0.74-1.25)	1.13 (0.85-1.50)	0.98 (0.73-1.31)	0.67 (0.48-0.94)*
**Gender**				
Male	1.00	1.00	1.00	1.00
Female	0.60 (0.50-0.72)^***^	1.02 (0.83-1.24)	1.19 (0.96-1.47)	1.15 (0.90-1.46)
**Location of tumor**				
Proximal colon	1.00	1.00	1.00	1.00
Distal colon	1.64 (1.30-2.08)^***^	1.39 (1.06-1.81)*	0.60 (0.47-0.76)^***^	0.88 (0.35-2.23)
Rectum	1.33 (1.05-1.67)*	2.25 (1.74-2.89)^***^	0.24 (0.18-0.32)^***^	0.78 (0.58-1.06)
not specified	0.76 (0.37-1.54)	1.43 (0.63-3.23)	1.20 (0.59-2.46)	0.88 (0.65-1.20)
**T-stage**				
T1-2	1.23 (0.86-1.76)	1.00 (0.71-1.42)	0.42 (0.23-0.77)^**^	1.17 (0.75-1.84)
T3	1.00	1.00	1.00	1.00
T4	0.48 (0.38-0.61)^***^	1.10 (0.84-1.43)	2.18 (1.72-2.77)^***^	1.28 (0.94-1.74)
not specified	1.14 (0.74-1.75)	1.32 (0.87-2.01)	1.26 (0.82-1.95)	3.76 (2.38-5.95)^***^
**N-stage**				
N0	1.00	1.00	1.00	1.00
N1	1.13 (0.90-1.41)	1.25 (0.99-1.59)	1.14 (0.87-1.49)	1.97 (1.43-2.72)^***^
N2	1.23 (0.95-1.58)	0.92 (0.70-1.22)	1.40 (1.05-1.87)*	3.03 (2.14-4.29)^***^
**Histology**				
Non-mucinous adenoca	1.00	1.00	1.00	1.00
Mucinous adenoca	0.46 (0.34-0.62)^***^	0.74 (0.52-1.05)	2.53 (1.84-3.47)^***^	1.20 (0.82-1.77)
**Onset of metastases**				
Synchronous	1.00	1.00	1.00	1.00
Metachronous	0.38 (0.31-0.47)^***^	4.10 (3.31-5.09)^***^	1.09 (0.86-1.37)	4.35 (3.31-5.71)^***^

In multivariate analyses adjustments were made for age, gender, location of the primary tumor, primary tumor stage, primary lymph node stage, tumor histology and onset of metastases.

* *p* < 0.05

** *p* < 0.01

*** *p* < 0.001.

Abbreviations: MV: multivariate; OR: Odds Ratio; adenoca: adenocarcinoma.

### Distant lymph node metastases

**Figure 2:**
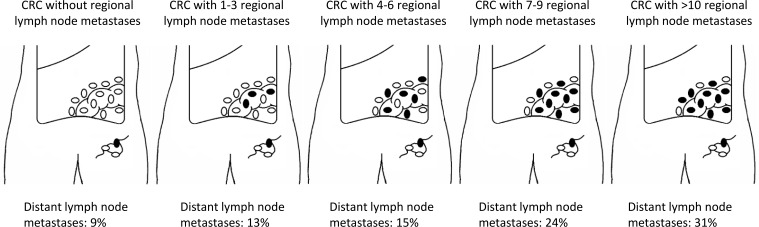
Percentage of patients with distant lymph node metastases according to the number of positive lymph nodes in primary tumor Selection of patients with more than ten lymph nodes examined (*N* = 1024 pts; autopsy cohort = 258 pts; clinical cohort = 766 pts), *p* < 0.001.

To provide further insight into the relevance of regional lymph node metastases for distant lymph node metastases, data was analyzed from 1024 cases with at least ten lymph nodes retrieved with the primary tumor. We found an increasing rate of distant lymph node metastases according to the number of positive lymph nodes detected in the primary tumor (Figure 2). Data from the ECR showed an increase from 9.1% in patients without positive lymph nodes to 27.1% in patients with more than twelve positive lymph nodes detected in the primary tumor (p<0.001). Data from the autopsy study showed an increase from 8.2% to 48.4% (p<0.001).

Compared with patients without distant lymph node metastases, patients with distant lymph node metastases more often had metastases in lung (57% vs. 29%, p<0.001), pleura (11% vs. 3%, p<0.001), bone (16% vs. 4%, p<0.001), adrenal gland (16% vs. 5%, p<0.001) and kidney (7% vs. 2%, p<0.001) (Figure 1b, Supplementary Figure 1b).

## DISCUSSION

This is the first large study comparing metastatic patterns according to lymph node status of CRC. The most common site of distant metastasis was liver followed by lung, peritoneum and distant lymph nodes, with percentages comparable to literature.[2, 6] Peritoneal and distant lymph node metastases occurred more often in regional lymph node positive CRC, while liver and lung metastases occurred in a similar percentage. Our multivariate analysis shows that next to established risk factors for peritoneal carcinomatosis, like T-stage, proximal location and mucinous carcinoma, regional lymph node metastases are an important risk factor. This is in line with other studies [7–10] making it likely that lymph node metastases are involved in the etiology of peritoneal carcinomatosis. The omentum is a preferential site of peritoneal metastases and the lymphoid milky spots in the omentum are a homing site for metastatic cancer cells.[11] Tumor cells in the omentum can reach the peritoneal cavity by direct growth. The milky spots and the anti-inflammatory function of the omentum suggest a relation between the peritoneum and the lymphatic system. Moreover, chylous ascites can occur after obstruction or resection of extra-peritoneal located lymphatic vessels, providing more evidence for direct communications between lymphatic vessels and the peritoneal cavity.[12] The finding that regional lymph node positive CRC spread more often to distant lymph nodes is in line with others [5] and in support of the first part of the lymphatic hypothesis. However, since no difference was observed in liver metastases, the vascular hypothesis seems more important for liver metastases.[4] Based on the lymphatic hypothesis we expected that lymphatic drainage of the thoracic duct into the venous system would lead to a higher incidence of lung metastases in regional lymph node positive CRC. However, we found comparable incidences of lung metastases in the whole group of patients with regional lymph node positive CRC (n = 1590). In a subgroup of patients with *distant* lymph node metastases analysed in the autopsy cohort (n = 297), we did find increased lung and pleural metastases, suggesting that for this small subgroup the lymphatic pathway is important for the metastatic pattern. Our unique setup, where we validated findings from autopsy studies in a registry based cohort with prospectively collected data, illustrated only a limited influence of lymph node metastases on metastatic patterns in CRC. Autopsy studies contain selected populations, in which patients are included who have died postoperatively, had an unexpected clinical course, or died of other causes than CRC. Nevertheless, autopsy studies offer a unique opportunity to study the final distribution of metastases. During autopsy all intra-abdominal and intra-thoracic organs are extensively explored, revealing more metastases than would have been detected with imaging. This explains the high rate of distant lymph node metastases found in the autopsy cohort compared to the clinical cohort. 17% of cases (286/1679) in the autopsy cohort and 22% of cases (673/3092) in our clinical cohort had to be excluded because of non-documentation of the regional lymph node status, since there was no resection of the primary tumor. This might have caused bias in our patient selection. Moreover, there could have been variations in the quality of the autopsy examination and in the pathological examination of resected primary tumor specimens, which will have occurred in both groups. Therefore we do not expect a significant bias. We cannot exclude the possibility that changes in the management of colorectal cancer between 1991-2010, would have had an influence on the metastatic patterns established at autopsy. The introduction of radiotherapy, chemotherapy, targeted therapy and surgery of metastatic lesions, might have shifted the presence of metastases to more uncommon sites. However, our main findings were validated in the population study with a narrower time frame. This study shows that regional lymph node involvement in CRC is associated with a higher rate of peritoneal metastases and distant lymph node metastases. Our findings support the hypothesis that metastases to the liver and lung occur independently of lymphatic spread. Regional lymph node metastases function as a biomarker, i.e. as a sign of advanced disease, and seem only mechanisticly involved in the process of metastases in a small subgroup of patients with spread via the distant lymph nodes. Unfortunately, the presence of vascular invasion is grossly underreported in pathology reports, making a separate analysis for vascular invasion not possible. Therefore, our findings can only indirectly support the vascular pathway as a mechanism for development of common distant metastases, such as liver and lung metastases. However, the current recognition of extramural vascular invasion in the staging and treatment of colorectal cancer [13–15] seems justified.

## MATERIALS AND METHODS

### Study design

An autopsy cohort was selected to compare patterns of metastases according to the regional lymph node status of the primary tumor. Findings at autopsy are the ultimate endpoint of disease, making autopsy reports suitable for analyzing the extend of disease. Autopsy data are derived from a restricted patient population, therefore a prospectively collected cohort of the Eindhoven Cancer Registry (ECR) was chosen for validation. First, differences in metastatic pattern according to the lymph node status of the primary tumor were analysed. Due to the close relationship between lymphatic spread, regional and distant lymph node metastases, separate analyses were performed in tumors with and without distant lymph node metastases in the autopsy cohort.

### Autopsy cohort

A total of 1679 patients with metastatic colorectal cancer was identified in an autopsy study by Hugen et al.[16] Data from this study was used for the present analyses. Patients were selected from a retrospective review of pathological and autopsy records from the nationwide network and registry of histo- and cytopathology in the Netherlands (Pathologisch-Anatomisch Landelijk Geautomatiseerd Archief; PALGA)[17] Patients who were diagnosed with metastatic CRC and autopsied between 1991 and 2010 were selected. In the Netherlands post-mortem examination is performed at the request of the family or treating doctor and is carried out by a pathologist. All autopsies included in this study were performed in order to obtain information on the medical status of the deceased or to determine the exact cause of death. No forensic autopsies were included. Tumor histology was assessed by different pathologists. Local staging of the primary tumor was reconstructed according to the TNM classification (5^th^ edition).[18] Allocation to the lymph node category was based on pathological examination of the original resection specimen or on the autopsy specimen. For regional lymph nodes metastasis only positive lymph nodes along the colon or rectum, plus the nodes along the major arteries that supply blood to the colon or rectum were considered. Metastasis in all other nodes were considered distant lymph nodes metastases. Patients of whom the primary N-stage could not be retrieved were excluded (n=286). Colon tumors were classified as proximal if they were found in cecum, ascending colon or transverse colon, and classified as distal if they were found in the descending or sigmoid colon. Data on gender and age were available for all cases, but further clinical information (e.g. treatment or disease course) was lacking in this database and could not be retrieved. Metastatic disease was determined during pathological assessments of resected or biopsied specimens during follow-up or during autopsy. All metastases found at autopsy were histologically confirmed. Metastases that were detected more than six months after surgery of the primary tumor were considered metachronous.[19]

### Clinical cohort

Data were retrieved from the Eindhoven Cancer Registry (ECR) which collects data of all patients with newly diagnosed cancer in the southeastern part of the Netherlands.[20] All patients who were diagnosed with CRC between 2003 and 2008 were included if they had synchronous metastases or developed metastases during follow-up untill 2010-2011 (n=3092). End of follow-up was defined as the date of death or end of data collection in 2010-2011. Patients who underwent an autopsy were excluded to prevent overlap with the initial cohort (n=37). Moreover, patients with missing N-stage (n=673) were excluded. Tumor staging, classification of primary tumor location and onset of metastatic disease were performed as described for the autopsy cohort. Allocation to the lymph node category was based on pathological examination of the original resection specimen. Anatomical sites of metastases were registered according to the International Classification of Diseases for Oncology (ICD-O).[21] Patterns of metastatic disease were determined based on the first site of metastasis.

### Statistical analysis

The χ² test was used to compare baseline characteristics between regional lymph node positive and negative CRC. Logistic regression analysis was used to analyze patient and tumor characteristics associated with location specific metastases. This analysis was performed in the clinical cohort, because of potential bias in the autopsy cohort. Odds ratio's (ORs) were provided with their 95% confidence interval (CI). In multivariate (MV) analyses adjustments were made for age, gender, primary T-stage, primary N-stage, differentiation grade of primary tumor, localization of primary tumor, primary tumor histology and onset of metastases. Statistical analyses were performed using SAS/STAT1 statistical software (SAS system 9.3, SAS Institute, Cary, North Carolina, USA) and the statistical software package SPSS 20.0 (SPSS Inc, Chicago, Illinois, USA). All tests of significance were two sided and differences at P-values of ≤0.05 were considered to be significant.

## SUPPLEMENTARY MATERIAL FIGURES


