# Supplementary material for: Limited effect of lymph node status on the metastatic pattern in colorectal cancer
Source: Oncotarget. 2016 Apr 27;7(22):31699–707. doi: 10.18632/oncotarget.9064 (PMC5077970; doi:10.18632/oncotarget.9064)
Supplement: Supplementary file 1 [file oncotarget-07-31699-s001.pdf]

Limited effect of lymph node status on the metastatic pattern in colorectal cancer

Supplementary Material

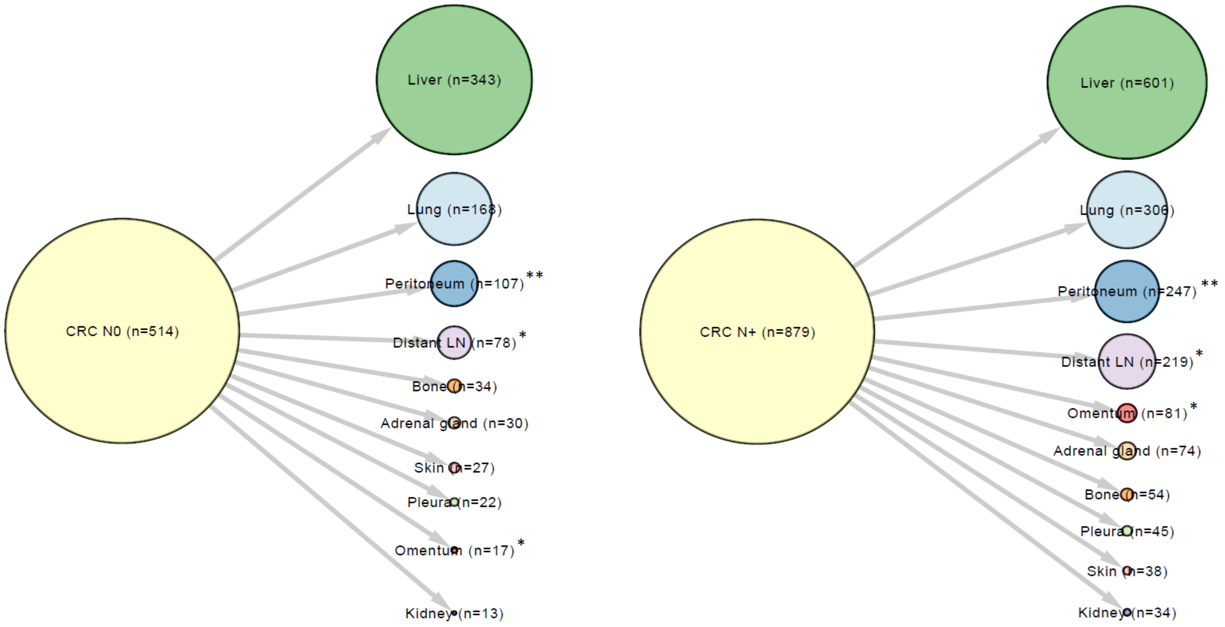

Supplementary Figure 1a. Distribution of CRC metastases according to regional lymph node status in the autopsy cohort.

Clock plots were made, using Cytoscape version 3.2.1. Only metastases with a frequency of 5% or higher were included in the clock plots. Left clock plot shows the distribution of metastases for regional lymph node negative primary tumors, right clock plot shows the distribution of metastases for regional lymph node positive primary tumors. Circle size is proportional to the percentage of metastases. * p=0.003, ** p<0.001

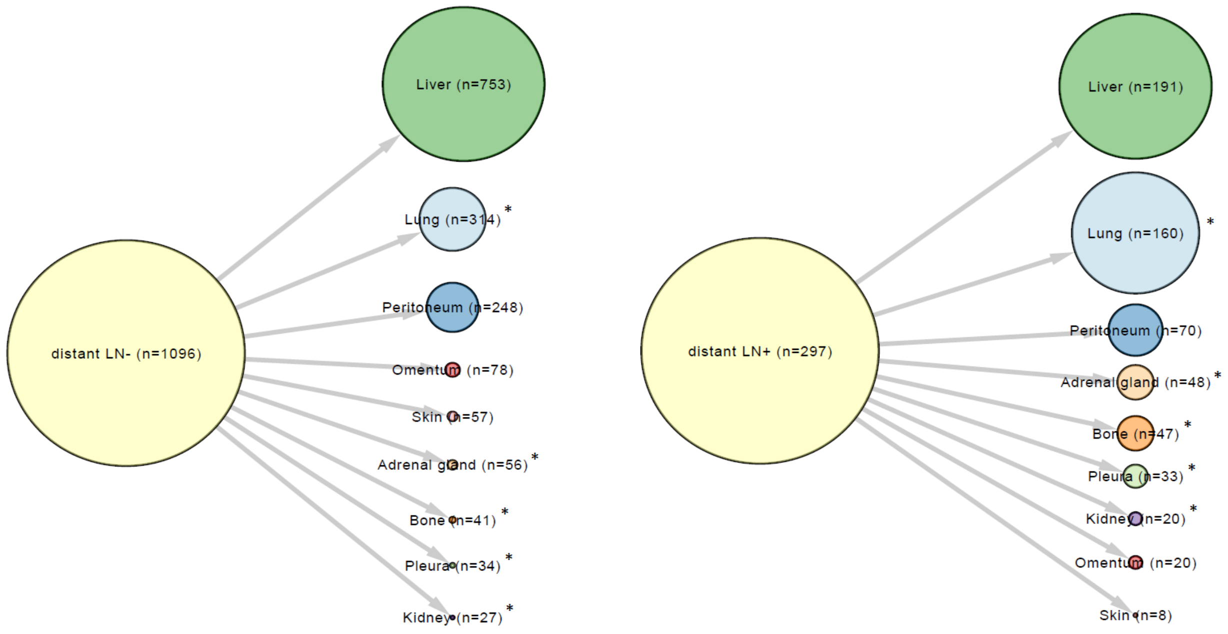

Supplementary Figure 1b. Distribution of CRC metastases according to distant lymph node positivity in the autopsy cohort.

Clock plots were made, using Cytoscape version 3.2.1. Only metastases with a frequency of 5% or higher were included in the clock plots. Left clock plot shows the distribution of metastases for primary tumors without distant lymph node metastases, right clock plot shows the distribution of metastases for primary tumors with distant lymph node metastases. Circle size is proportional to the percentage of metastases. * $p<0.001$
